# A Diamond Test

**Published:** 1870-01

**Authors:** 


A Diamond Test.—The more valuable an article is the more it is counterfeited, and the greater the perfection to which falsification is carried. The diamond has beeu so successfully imitated that he must be an expert indeed who can tell the false from the true. It by no means follows that because a man deals in jewelry his honesty must be of the first water, and the fact of a purchaser having paid for a diamond is not always proof that he has obtained one. There are known tests of genuineness, it is true; but they are chiefly optical, and require apparatus and skill to make them. A method which any one can apply, or easily get applied, has been a desideratum; but the want exists no longer. If you have a doubtful stone, put it, or cause it to be put, into a leaden or platinum cup, with some powdered fluor-spar and a little oil of vitrol; warm the vessel over some lighted charcoal, in a fire-place, or wherever there is a strong draught, to carry away the noxious vapors that will be copiously evolved. When these vapors have ceased rising let the whole cool, and then stir the mixture with a glass rod to fish out the diamond. If you find it intact it is a genuine stone; but if it is false it will be corroded by the hydrofluoric acid that has been generated around it. A small "paste" diamond would disappear altogether under this treatment. They who profit by this recipe have to thank Signor Massimo Levi, an Italian chemist.—*Am. Gas Light Journal.*

